# A Metabolomics Study on the Bone Protective Effects of a Lignan-Rich Fraction From Sambucus Williamsii Ramulus in Aged Rats

**DOI:** 10.3389/fphar.2018.00932

**Published:** 2018-08-21

**Authors:** Hui-Hui Xiao, Tung-Ting Sham, Chi-On Chan, Meng-Heng Li, Xi Chen, Qing-Chang Wu, Daniel Kam-Wah Mok, Xin-Sheng Yao, Man-Sau Wong

**Affiliations:** ^1^State Key Laboratory of Chinese Medicine and Molecular Pharmacology (Incubation), Hong Kong Polytechnic University Shenzhen Research Institute, Shenzhen, China; ^2^Department of Applied Biology and Chemical Technology, Hong Kong Polytechnic University, Hong Kong, China; ^3^School of Biotechnology, East China University of Science and Technology, Shanghai, China; ^4^Institute of Traditional Chinese Medicine & Natural Products, Jinan University, Guangzhou, China

**Keywords:** metabolomics, lignan, anti-osteoporosis, Sambucus Williamsii Ramulus, amino acid metabolism, anti-oxidative action

## Abstract

The lignan-rich fraction (SWR) of Sambucus Williamsii Ramulus, a folk herbal medicine in China for treatment of bone diseases, has previously reported to exert protective effects on bone without exerting uterotrophic effects in ovariectomized (OVX) mice. The aim of the present study was to identify the potential metabolites and the associated metabolic pathways that contribute to the beneficial effects of SWR on bone *in vivo*. Aged female Sprague Dawley rats (9 months old) were either sham-operated or ovariectomized for 12 weeks, before receiving treatment for another 12 weeks with the following treatment groups (*n* = 12 each): vehicle (Sham), vehicle (OVX), Premarin (130 μg/kg) or low (57 mg/kg), medium (114 mg/kg), and high (228 mg/kg) doses of SWR. The results showed that SWRH significantly suppressed bone loss, improved bone micro-architecture and increased bone strength on tibia without stimulating uterus weight gain in OVX rats. Premarin exerted similar bone protective effects as SWRH but elicited uterotrophic effects in OVX rats. The metabolic profiles of serum samples were analyzed by using ultra-performance liquid chromatography quadrupole time-of flight mass spectrometry and gas chromatography time-of flight mass spectrometry, and the metabolites that were significantly altered were identified by multivariate statistical analysis. Our study indicated that SWRH effectively restored the changes of 26 metabolites induced by estrogen-deficiency in OVX rats, which related to lipids, amino acids, tryptophan metabolisms, and anti-oxidative system. A subsequent validation showed that the serum level of superoxide dismutase and catalase were indeed up-regulated, while the serotonin level in a tryptophan hydroxylase 1 (TPH1) high expressing cells (rats RBL-2H3 cells) was down regulated after treatment with SWR. The results also suggested that the gut-microbiota may play an important role on the bone protective effects of SWR. The current study provides insight for understanding the unique mechanism of actions of SWR that might be involved in achieving bone protective effects *in vivo*.

## Introduction

Osteoporosis, characterized as the reduction of bone mass and deterioration of bone architecture, is a major cause of morbidity and mortality in the aging population. Worldwide, osteoporosis causes more than 8.96 million fractures in the population of age over 50 years in 2000 (Johnell and Kanis, 2006) and up to 50% of women and 20% of men >50 years of age are at risk of fractures (Eastell et al., 2016; Curtis et al., 2017). Metabolic pathways play an important role in age-related bone loss. Several circulating metabolites and proteins have been developed to be biomarkers for identifying the state of bone metabolism that aimed for diagnosis and treatment of bone diseases (Naylor and Eastell, 2012). Recently, the skeleton was considered as an endocrine organ and its metabolism has been demonstrated to be interacting with endocrine, inflammatory, immune, nutritional, gastrointestinal and renal systems (Baker-Lepain et al., 2011; Gimble, 2011; Gérard and Mathieu, 2012). Therefore, identification of metabolites that associated with bone loss will increase our ability to understand the biological mechanism of osteoporosis and to delineate the systematic actions of drug treatment.

Currently, commercially available drugs for treatment of osteoporosis include bisphosphonates, estrogen, selective estrogen receptor modulators (SERM), calcitonin, recombinant human parathyroid hormone (Ponnapakkam et al., 2013) and monoclonal antibody RANKL (Cummings et al., 2009). However, the long-term use of these drugs are compromised by the potential adverse effects, such as the increase in risk of cancers in reproductive system (Writing Group for the Women's Health Initiative Investigators, 2002), as well as high expenses and low drug tolerance (Stevenson et al., 2005). Alternative approach, such as traditional Chinese medicine, with long history of clinical application might offer a more acceptable and safer solution for management of osteoporosis.

We previously reported that 50 and 95% ethanol eluates from the 60% ethanol crude extract of Sambucus Williamsii Ramulus could improve bone properties in ovariectomized mice (Xiao et al., 2011; Zhang et al., 2011). Sambucus Williamsii Ramulus is the dried branches and stems of *Sambucus williamsii* Hance. In Chinese it named *JieGuMu* which literally means the bone connecting wood and it has been used in treatment of bone and joint-associated diseases ranging from wounds, bone fractures, rheumatoid arthritis, gout and Kaschin-Beck disease in China for thousands of years (Song, 2000; Xiao et al., 2016). In addition, it also used for relieving swelling and pain, promoting blood circulation, and acting as an anti-inflammatory agent in clinic (Xiao et al., 2011). Lignans have been identified to be the major and bioactive components in the active fraction, SWR (Xiao et al., 2014a, 2016). Although the protective effects of the lignan-rich fraction on bone had been demonstrated in adult mice, their therapeutic effects on aged animals have not been studied. More importantly, the systemic actions of lignan-rich fraction involving in bone protection *in vivo* are far from clear.

Metabolomics is a desired approach for studying the “global” change in metabolite profile in a biological system. The qualitative and quantitative measurement of endogenous metabolites could provide insights for understanding the biological changes that occurred in the internal environment in response to exogenous stimulation (Nicholson and Lindon, 2008). This approach is recently widely used in identification of potential biomarkers for disease diagnosis (Newgard, 2017) as well as characterization and qualification of traditional Chinese herb and formula (Ogegbo et al., 2012). It also serves as a powerful tool for assessing the therapeutic effects of herbal medicines and elucidating their underlying mechanism (Hu and Xu, 2014; Hang et al., 2015; Wang et al., 2017).

Herein, this study employed aged rat model with long-term ovary removal to mimic the conditions of patients suffering from established postmenopausal osteoporosis. SWR fraction with different dose and Premarin (conjugated estrogens) were supplemented upon the establishment of long term estrogen-deficient model. A metabolomics platform, using both ultra-performance liquid chromatography quadrupole time-of flight/mass spectrometry (UPLC-QTOF/MS) and gas chromatography time-of flight/mass spectrometry (GC-TOF/MS), was applied to obtain a holistic picture of the metabolic changes in serum of rats induced by ovariectomy and drug treatment. Validation study was carried out to confirm the results obtained from the metabolic profiles and to provide insights into understanding the mechanism of actions of the bone protective effect of SWR.

## Materials and methods

### Plant material and preparation of SWH sub-fraction

The stem branches of *S. williamsii* were collected in Caohekou Town, Benxi City, Liaoning Province in the northeast of China in July 2011 and authenticated according to a method listed in *Chinese Bencao* with the help of Professor Jincai Lu (Shenyang Pharmaceutical University, Liaoning, China). A voucher specimen (JGMGZ-2011) was deposited in the Herbarium of Pharmacy College, Jinan University (Guangzhou, China). The active sub-fraction of SWR was prepared followed the procedure described in our previous study (Xiao et al., 2011).

### Chemical and reagent

Uric acid, L-tryptophan, L-alanine, L-valine, L-leucine, L-isoleucine, glycine, l-serine, succinic acid, fumaric acid, malic acid, 2-ketoglutaric acid, L-glutamic acid, D-ribose, sodium 2-hydroxybutyrate, 3-hydroxybutyric acid, 2-isopropylmalic acid, formic acid, serotonin, γ-aminobutyric acid-2,2,3,3,4,4d6, 21-deoxycortisol, 11-deoxycortisol, oleic acid, cis-4,7,10,13,16,19-docosahexaenoic acid, cis-10-nonadecenoic acid (C19:1n9c) and sodium taurochenodeoxycholate were purchased from Sigma-Aldrich (St. Louis, MO, USA). Taurocholic acid was obtained from Steraloids (Newport, RI, USA). *p*-Cresyl sulfate potassium salt and arachidonic acid were supplied from Toronto Research Chemicals (North York, ON, Canada). Urea, HPLC-graded acetonitrile, methanol and isopropanol were obtained from Fisher Scientific (Hampton, NH, USA). Water was purified in-house using a Milli-Q Advantage A10 water purification system (Millipore, Bedford, MA, USA). Palmitic acid, cis-9,12-linoleic acid, 2-aminobutyric acid, pyridine (HPLC grade) with AcroSeal and N-methyl-N-(trimethylsilyl) trifluoroacetamide (MSTFA) were acquired from Acros Organics (Morris Plains, NJ, USA).

### Animal study

Female, retired breeder 9-month-old Sprague-Dawley rats were purchased from Vital River Company (Peking, China). The care and treatment protocol of this animal study was approved by the Animal Ethics Committee of the Hong Kong Polytechnic University. During the study, all rats were housed in the standard condition, allowed free access to distilled water and pair-fed the phytoestrogen-free diet (D00031602, Research Diet, NJ, USA) as reported in our previous study (Zhang et al., 2011).

Rats (*n* = 84) were divided randomly into two groups and subjected to sham operation (Sham, *n* 18) or ovariectomy (OVX, *n* = 66). Twelve weeks after surgery, the proximal tibias of six rats from each Sham and OVX group were scanned by Micro-CT to confirm the success of post-osteoporotic model. The remained rats were randomly divided to 12 rats per group and were oral administrated with either vehicle (Sham), vehicle (OVX), Premarin (PR, 130 μg/kg), low dose of SWR (SWRL, 57 mg/kg), medium dose of SWR (SWRM, 114 mg/kg) and high dose of SWR (SWRH, 228 mg/kg) for 12 weeks. The dosages of SWR were based on our previous experiment (Xiao et al., 2011). The dosage of Premarin (Wyeth, PA, USA) was calculated based on the clinical effective dose (1.25 mg conjugated estrogens/day). At the end of the treatment, urine was collected for 24 h and blood was withdrawn from abdominal aorta and serum was prepared and stored at −80°C. The uterus was collected and weighed, and the left femur and tibia were dissected and wrapped in PBS buffer for further analysis.

### Chemistries and enzymes in serum and urine

Calcium and phosphorus levels in serum and urine were measured by using commercial kits (STANBIO Laboratory, Texas, USA). Serum osteocalcin was measured using Rat-MID™ Osteocalcin ELISA kit (Immumodiagnostic Systems Ltd, Boldon, UK). Urinary creatinine (Cr) used as internal control was assessed by using a commercial kit (STANBIO Laboratory, Texas, USA). Urinary calcium and phosphorus levels were expressed as its excretion per unit of Cr (Ca/Cr, P/Cr).

The oxidative stress marker malondialdehyde (MDA) was measured by thiobarbituric acid method according to procedure described in a commercial kit (KegGen BioTech, Nanjing, China). The activities of superoxide dismutase (SOD) were determined by inhibiting the production of superoxide anion free radical in xanthine-xanthine oxidase system according to the manufacturer's instruction (KegGen BioTech, Nanjing, China). Catalase (CAT) activity was determined by its ability of decomposing H_2_O_2_ according to the manufacturer's instruction (KegGen BioTech, Nanjing, China).

### Micro-computed tomography (micro-CT) analysis of bone properties

Bone properties at the proximal metaphysis of tibia were measured by using Micro-CT (VivaCT 40, Scanco Medical, Brassdrof, Switzerland). The starting scan site was defined as 2.0 mm away from the tibia head. The bone samples were scanned in the axial direction with a resolution of 21 μm and a scanning power of 70 kVp and 110 μA. Twenty consecutive slices (length of 0.42 mm) were used for contouring the volume of interest (VOI) to evaluate the morphological properties of tibia. The threshold values for contouring VOIs was 200, which were based on the contoured image matched with the grayscale of the background image (Wong et al., 2014). Contoured VOI images were evaluated by the Evaluation Program v6.0 (Scanco) to generate 3D bone biological parameters as follow: bone mineral density (BMD), trabecular bone number (Tb.N), trabecular bone separation (Tb.Sp), bone volume over total volume (BV/TV), Connectivity density (Conn.D), and structure model index (SMI).

### Biomechanical measures of tibia mid-diaphysis

The biomechanical properties of mid-shaft tibia were measured using specified three-point bending machine (Hounsfield Test Equipment Limited, Surrey, UK) (Pang et al., 2010). The outer two supporting points were fixed 15 mm apart with a single central loading pressure at a deformation rate of 2.0 mm/min and a load-deformation curve was plotted simultaneously until the specimen was broken. Bone mechanical parameters such as yield stress (YLD.STR), yield displacement (YLD.DISP), ultimate load and modulus were exhibited.

### Serotonin biological assay *in vitro*

RBL-2H3 cells (TPH-1 expressing cells, purchased from Procell, Wuhan, China) were routinely cultured in minimum essential medium (MEM) spplemented with 15% fetal bovine serum (FBS), penicillin 100 U/ml and steptomycin 100 μg/ml (Invitrogen, CA, USA) at 37°C in a humidified atmosphere of 95% air and 5% CO_2_. The cells were seeded in 96-well plates, 12-well plates and 6-well plates at a density of 1 × 10^4^, 1 × 10^5^, and 2 × 10^5^ per well, respectively. Cells were treated with SWR (0.1–100 μg/ml) for 48 h. Then MTS [3-(4,5-dimethylthiazol-2-yl)-5-(3-carboxy-methoxyphenyl)-2-(4-sulfo phenyl)-2H-tetrazolium] assay was used as an indirect colorimetric measurement of cell viability as previously described (Xiao et al., 2014b).

Treated cells were lysed, centrifuged and the concentration of serotonin in the supernatant was measured by using commercial kit (Signalway Antibody, USA). The protein of treated cells was extracted and subjected to sodium dodecyl sulfate polyacrylamide gel electrophoresis (SDS-PAGE) and transblotted to polyvinylidene difluoride (PVDF) membranes (Immobilin-P, Millipore Corp., Bedford, MA, USA) as previously described (Xiao et al., 2014b). The blots were probed with rabbit monoclonal TPH-1 (1:500; OriGene Tchnologies, Inc) and β-actin (1:5,000; Abcam). This was followed by incubation with goat anti-rabbit (1:2,000; Santa Cruz Biotechnology) conjugated with horseradish peroxidase. The antigen-antibody complexes were then detected with enhanced chemiluminescence (ECL) reagent (Pierce qb Perbio, Rockford, IL, USA) and visualized by the Lumi-Imager using Lumi Analyst version 3.10 software (Roche, Mannheim, Germany).

### Real-time quantitative reverse transcriptase-polymerase chain reaction (RT-PCR) analysis

Total RNA was extracted from the right tibia using TRIzol® reagent (Invitrogen, Rockville, MD, USA), and transcribed by using a high-capacity cDNA reverse transcription kit (Thermo Scientific, Lithuania, EU). Real-time PCR was performed in CFX 96 Real Time system (BioRad Laboratories, CA, USA). The PCR program was carried out as follow: denaturation 95°C for 1 min, amplification for 40 cycles (95°C for 20 s; 60°C for 20 s and 72°C for 18 s). The relative mRNA amount was normalized to GAPDH mRNA, a housekeeping gene. The PCR primers used in this study were listed in Table S1.

### Serum samples preparation

For LC/MS, 100 μl serum sample was mixed with 300 μl isopropanol [containing 100 ppm, cis-10-nonadecenoic acid (C19:1n9c) as internal standard] and vortexed for 30 s. Then, the mixture was stood at −20°C overnight for completion of deproteination. Then, 340 μl supernatant was collected after centrifugation at 18,700 g at 4°C for 20 min and dried under nitrogen gas. The dried supernatant was reconstituted in 100 μl of isopropanol-water (50:50, v/v) prior to UPLC-QTOF-MS analysis.

For GC-TOF/MS, 50 μL serum was mixed with 150 μL methanol (including 5 ppm 2-isopropylmalic acid as internal standard) and vortexed for 30 s. The mixture was stood at −20°C for 1 h for deproteinization. Then, 50 μl supernatant was collected after centrifugation at 18,700 g at 4°C for 20 min and dried under nitrogen gas. Subsequently, the dried supernatant was reconstituted in 75 μl methoxyamine hydrochloride in pyridine (15 mg/ml) for oximation of oxo-groups to methyloximes at 30°C under shaking for 1.5 h. Afterwards, 75 μL MSTFA was added and stood at 70°C for 1 h for silylation. The whole reaction was kept away from light. After the silylation, the resultant supernatant was transferred into an insert of amber vial for GC-TOF/MS analysis.

An equal volume of each serum sample was pooled, vortexed and aliquoted to provide pooled quality control (QC) samples and went through the same extraction protocols of LC-MS and GC-MS as described above like all other samples at each analytical batch. They were injected to monitor the stability of the instruments after every five-sample injection.

### UPLC-QTOF/MS and GC-TOF/MS analysis

The serum metabolites profiling was performed with a Waters ACQUITY UPLC system coupled with Waters SYNAPT G2 Q-IM-TOF HDMS system (Waters, Milford, USA) and an Agilent 7890A Gas-Chromatograph (Agilent Technologies Inc., CA, USA) coupled with Waters Micromass GCT Premier Time-of-Flight Spectrometer (Waters, Milford, MA, USA). Liquid chromatography analysis was carried out with a Waters ACQUITY UPLC HSS T3 column (2.1 × 50 mm, 1.8 μm) and Ailgent HP-5MS capillary column (30 m × 250 μm i.d, 0.25 μm), respectively. The chromatographic conditions and mass spectrometry parameters are listed in Supplementary Text S1.

### Data processing, identification of potential biomarkers, and construction of metabolic pathway

The peak picking, alignment and normalization of all raw UPLC–MS and GC-TOF/MS were conducted by Progenesis QI software (Nonlinear Dynamics, Newcastle upon Tyne, United Kingdom) and MarkerLynx application manager v 4.1 SCN 901 (Waters, Milford, USA), respectively. Data matrix was normalized by total ion abundance of its own sample (LC-MS) or internal standard (GC-MS) to generate a data matrix that consisted of the retention time, mass to charge ratio (m/z), and the intensity. Quality screening was done by filtering out those metabolites which relative standard deviation was greater than 30% in pooled quality control samples.

The identities of the specific metabolites were confirmed by comparison of their m/z, mass fragmentation patterns and chromatographic retention times with commercially available chemical standards or NIST library 2.0, online metabolite databases such as Human Metabolome Database (http://metlin.scripps.edu) and Metlin (http://metlin.scripps.edu) and literatures.

Pathway analysis and metabolite set enrichment analysis of significant biomarkers altered by SWRH treatment were performed with Metaboanalyst 4.0 (www.metaboanalyst.ca) for identification of the top altered pathways and visualization. Over-representation analysis was performed for the analyses using Fisher's exact test. Relative-betweenness centrality was used for the pathway topology analysis.

### Statistical analysis

The pharmacodynamic data was presented as mean ± SEM. Differences were analyzed statistically with one-way analysis of variance (ANOVA) followed by Tukey's *post*-*hoc* test or with unpaired Student's *t*-test using Graphpad PRISM® software package. *P* < 0.05 was considered statistically significance. The statistical analyses of the results from LC-MS and GC-MS were performed using SPSS PASW Statistics 18 (Chicago, IL, USA). After logarithmic transformation [log2 (normalized ion abundance) followed by removing outliers (1.5 times of the interquartile range), statistical differences were analyzed at a univariate level by ANOVA. Based on homogeneity of variances, for equal variance, Tukey's *post-hoc* test was used; for unequal variance, Tamhane's T2 *post-hoc* test was used. *P*-value ≤ 0.05 was regarded as statistically significant.

## Results

### Effects of SWR on body weight, uterus weight, biochemistry, bone mass, and bone related gene expression on rats

As shown in Table 1, upon pair-feeding, the body weight of rats in Sham group and Premarin group were decreased significantly compared with OVX group, while SWR did not significantly alter body weight in OVX rats. Premarin, but not SWR bioactive fraction, exert uterotrophic effects in OVX rats. SWR did not alter serum calcium, but significantly increased serum phosphorus and decreased urinary calcium and phosphorus excretion at mid and high dosages in OVX rats. In contrast, premarin only showed notable effects on inhibiting urinary calcium excretion in OVX rats. In addition, high dose of SWR significantly suppressed serum osteocalcin level in OVX rats.

**Table 1:** Bone parameters, body weight changes, uterine index, and levels of biochemical markers in serum and urine of rats.

			**Modeling for 12 weeks**		**Treatment for 12 weeks**					
			**Sham**	**OVX**	**Sham**	**OVX**	**PR**	**SWRL**	**SWRM**	**SWRH**
Bone parameters measured by Micro CT	Bone content	BMD (mgHA/cm^3^)	903.4 ± 8.6	373.7 ± 7.8^*###*^	300.4 ± 18.0	140.6 ± 13.8^*###*^	199.8 ± 12.6^**^	131.5 ± 11.5	172.6 ± 17.4	188.1 ± 17.4^*^
		Tb.N (mm^−1^)	4.14 ± 0.15	2.14 ± 0.23^*###*^	4.05 ± 0.10	1.99 ± 0.12^*###*^	2.70 ± 0.16^*^	2.22 ± 0.19	2.38 ± 0.11	2.60 ± 0.18^*^
		Tb.Sp (mm)	0.24 ± 0.01	0.51 ± 0.06^*##*^	0.14 ± 0.01	0.43 ± 0.03^*###*^	0.29 ± 0.02^**^	0.40 ± 0.02	0.36 ± 0.02	0.33 ± 0.02^*^
	Bone size	BV/TV (%)	0.204 ± 0.005	0.093 ± 0.017^*###*^	0.446 ± 0.029	0.176 ± 0.010^*###*^	0.254 ± 0.020^*^	0.189 ± 0.013	0.205 ± 0.016	0.245 ± 0.021^*^
	Bone structure	Conn.D (mm^−3^)	53.3 ± 3.65	13.7 ± 3.44^*###*^	83.9 ± 5.04	21.3 ± 2.24^*###*^	36.4 ± 3.23^*^	21.9 ± 1.79	27.0 ± 3.20	38.0 ± 5.57^*^
		SMI	2.01 ± 0.15	2.46 ± 0.13^#^	1.04 ± 0.29	1.95 ± 0.11^*##*^	1.26 ± 0.12^***^	1.67 ± 0.09	1.54 ± 0.07^*^	1.37 ± 0.08^**^
		Ultimate Load(N)	151.7 ± 9.0	125.0 ± 6.9^#^	148.4 ± 4.7	118.9 ± 3.5^#^	137.8 ± 5.5^*^	125.0 ± 6.5	135.1 ± 8.2	143.5 ± 6.8^*^
Biomechani–cal properties		DISP (mm)	0.55 ± 0.02	0.46 ± 0.03^#^	0.56 ± 0.03	0.40 ± 0.04^*##*^	0.52 ± 0.04^*^	0.48 ± 0.04	0.49 ± 0.05	0.53 ± 0.02^*^
		YLD.STR(Mpa)	217.2 ± 7.4	147.1 ± 5.1^*###*^	156.6 ± 5.0	126.3 ± 3.5^*##*^	151.4 ± 5.2^**^	138.6 ± 4.8	148.9 ± 4.0	152.6 ± 4.5^*^
		Modulus (N/mm^2^)	1732 ± 67	1357 ± 29^*###*^	1726.8 ± 90.5	1285.8 ± 49.8^*##*^	1718.6 ± 72.5^**^	1398.8 ± 70.7	1612.6 ± 88.5^*^	1774.0 ± 59.4^***^
Weight change (%)		/	/	/	−4.79 ± 1.56	0.68 ± 0.87^#^	−6.67 ± 2.01^*^	0.57 ± 1.12	0.35 ± 0.79	−1.40 ± 0.54
Uterus index (mg/g)		/	/	/	1.73 ± 0.16	0.49 ± 0.05^*###*^	1.16 ± 0.10^***^	0.51 ± 0.09	0.47 ± 0.06	0.53 ± 0.10
Serum Ca (mg/dl)		/	/	/	7.80 ± 0.24	7.78 ± 0.37	7.91 ± 0.26	8.41 ± 0.26	7.96 ± 0.22	8.19 ± 0.30
Serum P (mg/dl)		/	/	/	5.52 ± 0.26	4.67 ± 0.22^#^	4.50 ± 0.28	4.32 ± 0.25	5.69 ± 0.16^*^	6.05 ± 0.36^**^
Serum OCN (ng/ml)		/	/	/	108.7 ± 13.1	222.4 ± 20.3^*###*^	192.9 ± 5.7	194.1 ± 12.2	167.1 ± 13.4	156.2 ± 13.0^*^
Urine Ca/Cr (mg/dl)		/	/	/	0.18 ± 0.02	0.27 ± 0.03^#^	0.11 ± 0.02^***^	0.21 ± 0.03	0.16 ± 0.01^*^	0.15 ± 0.01^**^
Urine P/Cr (mg/dl)		/	/	/	0.18 ± 0.03	0.31 ± 0.05^#^	0.17 ± 0.04	0.22 ± 0.03	0.15 ± 0.02^*^	0.12 ± 0.04^**^

*/, undetermined parameter*.

*Nine-month old SD rats were subjected to the following treatment for 12 weeks started at 12 week after ovariectomy: Sham, Sham-operated, vehicle-treated; OVX, ovariectomized, vehicle-treated; PR, ovariectomized, Premarin-treated (0.13 mg/kg); SWRL, ovariectomized, low dose SWR-treated (57 mg/kg); SWRM, ovariectomized, mid dose SWR-treated (114 mg/kg); SWRH, ovariectomized, high dose SWR-treated (228 mg/kg). Results were expressed as mean ± SEM*.

### P < 0.001 vs. control;

* *P < 0.05*,

** *P < 0.01*,

*** *P < 0.001 vs. OVX*.

Ovariectomy for 24 weeks lead to continual loss of bone mineral density and deterioration of trabecular bone microarchitecture in rats as demonstrated by the reduction of Tb.N, BV/TV, and Conn.D and increase of Tb.Sp and SMI (Table 1). Treatment of the OVX rats with SWR attenuated the changes of trabecular bone properties in the OVX rats in a dose-dependent manner. The administration of high dose SWR and Premarin significantly reversed the ovariectomy-induced trabecular bone loss and microarchitecture deterioration in rats. OVX also significantly reduced the cortical bone strength in tibia in rats by decreasing ultimate load, yield displacement, yield stress and modulus. Treatment of OVX rats with Premarin and the high dosage of SWR could significantly inhibit the reduction of bone strength. SWR could increase bone strength in a dose-dependent manner, although the effects of low and mid dosages did not reach statistical significance.

**Figure 1:**
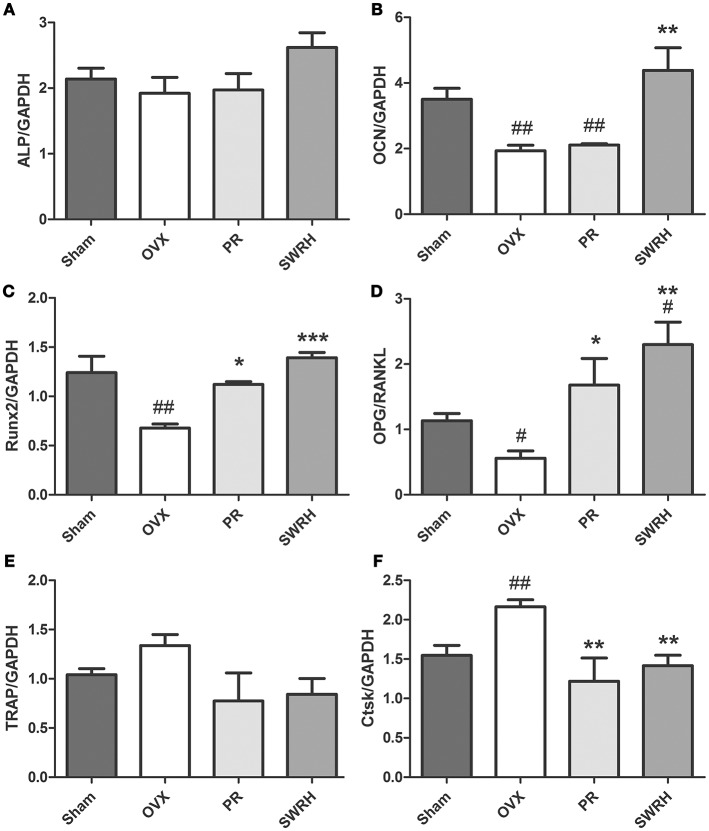
Effects of SWRH on bone specific mRNA expression in rat tibia. Nine-month old SD rats were subjected to the following treatment for 12 weeks started at 12 week after ovariectomy: Sham, Sham-operated, vehicle-treated; OVX, ovariectomized, vehicle-treated; PR, ovariectomized, Premarin-treated (0.13 mg/kg); SWRH, ovariectomized, high dose SWR-treated (228 mg/kg). **(A)** ALP, alkaline phosphatase; **(B)** OCN, osteocalcin; **(C)** Runx2, runt-related transcription factor 2; **(D)** OPG/RANKL, OPG, osteoprotegerin; RANKL, receptor activator of nuclear factor κB ligand; **(E)** TRAP, tartrate-resistant acid phosphatase; **(F)** Ctsk, cathepsin K; GAPDH, glyceraldehydes 3-phosphate dehydrogenase. Results were expressed as mean ± SEM. ^#^*P* < 0.05, ^*##*^*P* < 0.001 vs. control; *P < 0.05, **P < 0.01, ***P < 0.001 vs. OVX.

The effects of SWR on the mRNA levels of several osteoblast and osteoclast-related genes in bone obtained from OVX rats were studied (Figure 1). Treatment with high dosage of SWR enhanced the expression of specific markers involved in osteoblast differentiation, including Runx2, ALP, and osteocalcin (OCN). The system of osteoprotegerin (OPG) and receptor activator of nuclear factor-κB ligand (RANKL) which regulates bone resorption triggers the induction of osteoclast-specific genes, including cathepsin K and tartrate-resistant acid phosphatase (TRAP), and induces osteoclast differentiation and maturation (Kogawa et al., 2013). Treatment with high dosage SWR also inhibited the osteoclastogenesis by up-regulating the expression of the ratio of OPG/RANKL as well as down-regulating the expression of tartrate acid phosphatase (TRAP) and cathepsin K (Ctsk) genes. This data suggested the high dose SWR could exert beneficial effects on bone by modulating the process of both osteoblastogenesis and osteoclastogenesis.

Taken together, the animal experiments revealed that the lignan-rich fraction of *S. williamsii* is useful for maintaining bone health on both trabecular bone and cortical bone in aged ovariectomized rats without side effects of uterotropy. In addition, the mechanism underlying its effects include regulating the process of osteoblastogenesis and osteoclastogenesis.

### Data acquisition and identification of potential metabolic biomarkers

**Figure 2:**
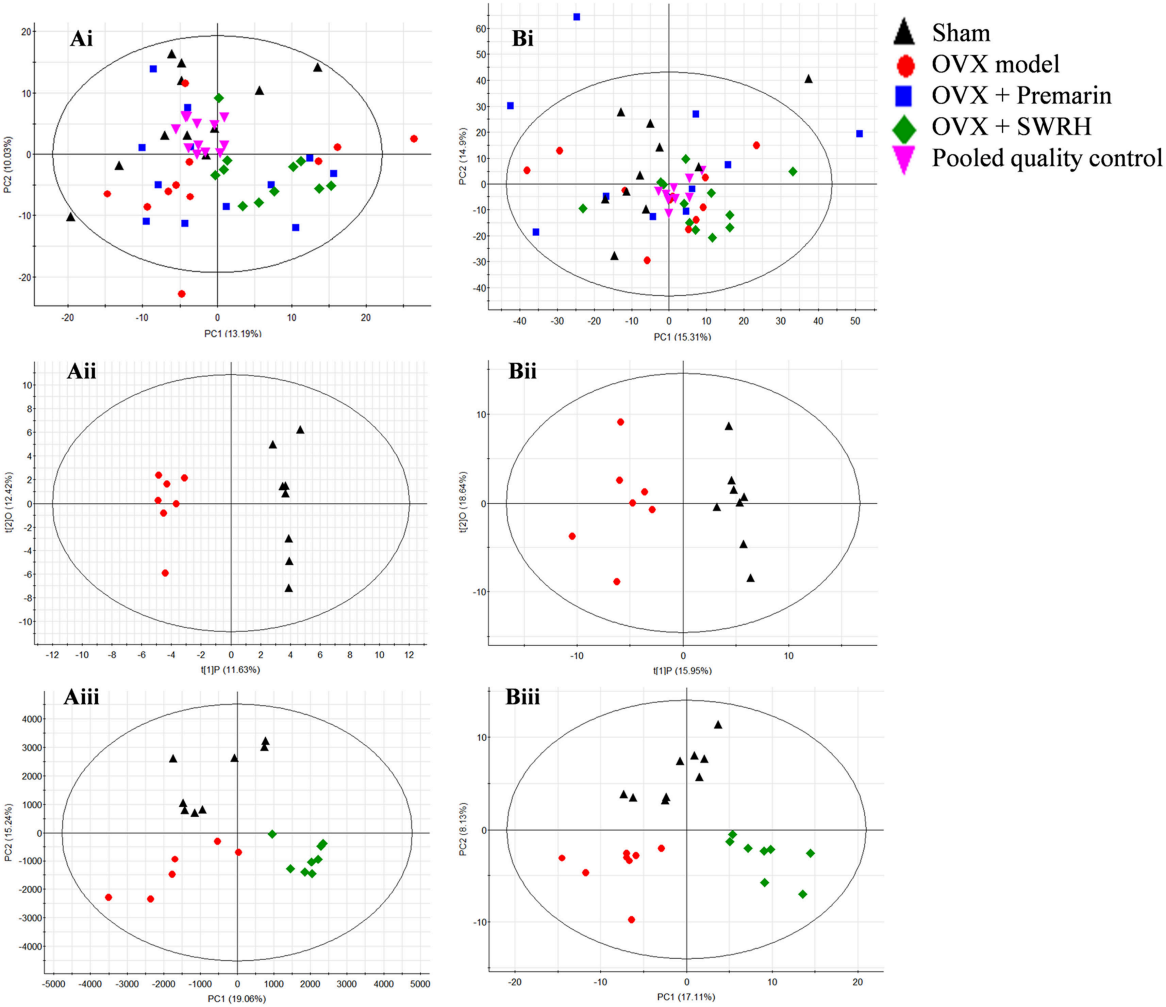
PCA, OPLS-DA, and PLS-DA score plots in UPLC-MS and GC-MS. (i) PCA score plot, (ii) OPLS-DA score plot, and (iii) PLS-DA score plot of metabolites for **(A)** UPLC-MS acquired in negative ESI mode and **(B)** GC-MS. OPLS-DA score plot: UPLC-MS, R^2^X _(cum)_ = 0.249, R^2^Y _(cum)_ = 0.962, Q^2^_(cum)_ = 0.558; GC-MS, R^2^X _(cum)_ = 0.346, R^2^Y _(cum)_ = 0.910, Q^2^_(cum)_ 0.688. PLS-DA score plot: UPLC-MS, R^2^X _(cum)_ = 0.343, R^2^Y _(cum)_ = 0.760, Q^2^_(cum)_ 0.599; GC-MS, R^2^X_(cum)_ = 0.252, R^2^Y_(cum)_ = 0.795, Q^2^_(cum)_ = 0.510.

The metabolic profile obtained from negative electrospray ionization (ESI) mode of UPLC-QTOF/MS and GC-TOF/MS were plotted using principal components analysis (PCA), orthogonal partial least squares discriminant analysis (OPLS-DA) and partial least squares discriminant analysis (PLS-DA) in Figure 2. PCA was used to explore general interrelations between groups, while PLS-DA was applied to maximize the difference of metabolic profiles and facilitate the detection of metabolites consistently present in the samples. A high degree of aggregation in all quality controls (QCs) in both LC-MS and GC-MS was observed in PCA score plot, indicating the good stability of the two analytical platforms throughout the entire experiment. An internal cross validation of EZinfo software was carried out to estimate the performance of PLS-DA and OPLS-DA models. The calculated cumulative values of R^2^X and R^2^Y estimates the goodness of fit of the model that represents the fraction of explained X- and Y-variation. The cumulative value of Q^2^ estimates the predictive accuracy of the model with threshold >0.5 and its difference from R^2^Y should not exceed 0.3 (Eriksson et al., 2013). For OPLS-DA, the cumulative R^2^X, R^2^Y, and Q^2^ value was 0.241, 0.982, and 0.705, respectively, in LC/MS, and 0.346, 0.910, 0.688, respectively, in GC-MS, indicating a robust separation of Sham and OVX group in the respective score plot. For PLS-DA, the cumulative R^2^X, R^2^Y, and Q^2^ value was 0.343, 0.760, and 0.599, respectively, in LC/MS, and 0.252, 0.795, 0.510, respectively, in GC-MS, validating the clear classification of Sham, OVX and SWRH groups in its score plot without overfitting.

From the score plot of OPLS-DA, 145 ions in LC-MS and 273 fragments in GC-MS were deemed discriminatory [threshold of variable importance in the projection (VIP) > 1] and identified as being responsible for the separation between Sham and OVX groups. Further identification of potential biomarkers was performed by comparing with reference standards or based on their molecular ion information of *m/z*, retention time and fragments. Ultimately, 51 ions (27 in LC-MS and 24 in GC-MS) that significantly changed with *P* < 0.05 between the Sham and OVX groups were identified as metabolic biomarkers and listed in Tables 2, 3. Among them, 29 metabolites were successfully matched with reference standards. Compared with Sham group, there are 22 down-regulated and 5 up-regulated metabolites in LC-MS, and 20 down-regulated and 4 up-regulated metabolites in GC-MS.

**Table 2:** Identified differential metabolites in serum accountable for the discrimination between OVX rats and Sham rats by UPLC-MS in negative ESI mode.

**Metabolites**	**Retention time (min)**	**Adducts**	**Theoretical m/z**	**Detected m/z**	**Mass error (ppm)**	**Fold change (normalized ion abundance)**		**Change trend compared with OVX group**		***P*****-value compared to OVX model**^**#**^	
						**Sham/OVX**	**SWRH/OVX**	**Sham**	**SWRH**	**Sham**	**SWRH**
Uric Acid^∧^	0.59	[M-H]-	167.0211	167.0205	−3.59	1.39	1.26	↑	↑	0.016^*^	0.039^*^
Succinic acid^^∧^^	0.75	[M-H]-	117.0193	117.019	−2.56	1.93	1.06	↑	–	0.007	0.926
Tryptophan^^∧^^	2.53	[M-H]-	203.0826	203.0821	−2.46	1.32	1.43	↑	↑	0.023	0.002
*p*-Cresyl sulfate^^∧^^	2.73	[M-H]-	187.0071	187.0065	−3.21	0.39	0.23	↓	↓	0.033	0.001
Taurocholate^^∧^^	3.23	[M-H]-	514.2844	514.2841	−0.58	0.24	0.37	↓	–	0.034	0.264
Sulfate metabolite 1	3.52	N/A	427.1796	427.1801	1.17	2.52	0.32	↑	↓	0.039	0.006
Taurochenodeoxycholate^^∧^^	3.77	[M-H]-	498.2895	498.2891	−0.80	0.37	0.26	–	↓	0.053	0.002
Unidentified 1	3.92	N/A	413.2003	413.2001	−0.57	11.96	0.48	↑	–	0.009^*^	0.079^*^
Unidentified 2	4.30	N/A	405.2646	405.2643	−0.86	6.85	1.16	↑	–	0.013	0.964
Unidentified 3	4.50	N/A	347.2228	347.2223	−1.39	9.49	1.81	↑	–	<0.001^*^	0.336^*^
Sulfate metabolite 2	4.86	N/A	455.2473	455.2471	−0.44	8.88	0.32	↑	–	0.003^*^	0.051^*^
Unidentified 4	4.89	N/A	349.2384	349.238	−1.24	12.28	0.44	↑	–	0.009	0.235
Deoxycortisol/isomer 1	5.00	[M-H]-	345.2071	345.2068	−0.87	3.87	0.85	↑	–	0.001	0.790
Deoxycortisol/isomer 2	5.18	[M-H]-	345.2071	345.2067	−1.16	5.20	0.17	↑	↓	0.024	0.013
LysoPC (20:4)	7.18	[M+FA-H]-	588.3307	588.3305	−0.34	1.24	1.14	↑	–	0.010	0.127
LysoPC (22:6)	7.23	[M+FA-H]-	612.3307	612.3306	−0.16	1.86	1.36	↑	↑	<0.001	0.013
LysoPE (18:2)	7.33	[M-H]-	476.2783	476.2779	−0.84	0.75	0.66	↓	↓	0.030	0.001
LysoPC (22:6)	7.64	[M+FA-H]-	612.3307	612.3307	0	1.58	1.30	↑	↑	<0.001	0.019
LysoPC (22:5)	8.79	[M+FA-H]-	614.3463	614.3463	0.49	3.58	1.60	↑	↑	<0.001	0.035
LysoPE (22:5)	9.14	[M-H]-	526.2939	526.2948	1.71	1.71	1.01	↑	–	<0.001	0.936
LysoPE (18:1)	9.31	[M-H]-	478.2939	478.2938	−0.21	0.80	0.80	↓	↓	0.018	0.018
LysoPC (22:4)	10.12	[M+FA-H]-	616.362	616.3619	−0.16	1.51	1.48	↑	↑	0.012	0.011
LysoPC (18:0)	10.77	[M+FA-H]-	568.362	568.3618	−0.35	1.13	1.13	↑	↑	0.005	0.003
Docosahexaenoic acid^^∧^^	12.35	[M-H]-	327.233	327.2326	−1.22	2.28	1.22	↑	–	0.002^*^	0.766^*^
Arachidonic acid^^∧^^	12.48	[M-H]-	303.233	303.2323	−2.31	1.28	1.20	↑	↑	0.001	0.018
Linoleic acid^^∧^^	12.56	[M-H]-	279.233	279.2325	−1.79	1.77	1.43	↑	↑	<0.001	0.012
Oleic acid^^∧^^	13.06	[M-H]-	281.2486	281.2483	−1.07	1.82	1.62	↑	↑	<0.001	0.004

*Nine-month old SD rats were sham operated or ovariectomized. At 12 week after ovariectomy, the rats were vehicle treated for 12 weeks. Log_2_ transformation of normalized ion abundance prior to one-way ANOVA test*.

# Equal variance: Tukey HSD;

* Unequal variance: Tamhane's T2 post-hoc test; N/A, not available; FA, formic acid;

∧ *Identified with authentic standards; ↑ upregulated vs. OVX group; ↓ downregulated vs. OVX group; –, no significant change*.

**Table 3:** Identified differential metabolites accountable for the discrimination between OVX rats and Sham rats by GC-TOF/MS.

**Metabolites**	**Retention time (min)**	**Mass fragment**	**Fold change (normalized peak area)**		**Change trend compared with OVX group**		***P*****-value compared to OVX model** ^**#**^	
			**Sham/OVX**	**SWRH/OVX**	**Sham**	**SWRH**	**Sham**	**SWRH**
Alanine^∧^	5.93	116.10	1.45	1.71	↑	↑	0.048	0.002
2-hydroxybutyric acid^∧^	6.28	131.10	1.85	0.66	↑	–	0.012	0.258
3-hydroxybutyric acid^∧^	6.72	233.12	2.98	1.54	↑	–	0.010	0.457
2-aminobutyric acid^∧^	6.9	130.11	2.38	1.03	↑	–	0.001	0.689
Valine^∧^	7.53	144.14	1.40	1.28	↑	↑	0.001	0.014
Urea^∧^	7.83	147.08	0.53	0.40	↓	↓	0.008	0.000
Leucine^∧^	8.31	158.15	1.33	1.31	↑	↑	0.010	0.015
Isoleucine^∧^	8.61	218.11	1.43	1.36	↑	↑	0.004	0.019
Glycine^∧^	8.78	248.15	0.70	1.25	↓	↑	0.002	0.030
Succinic acid^∧^	8.84	147.07	1.97	1.30	↑	–	0.021	0.368
Fumaric acid^∧^	9.27	245.07	1.48	1.37	↑	↑	0.003	0.018
Serine^∧^	9.54	204.14	1.52	1.54	↑	↑	0.019	0.005
Malic acid^∧^	11.18	233.11	2.03	1.13	↑	–	<0.001	0.719
Methionine^∧^	11.51	176.10	1.32	1.39	↑	↑	0.005	0.001
2-ketoglutaric acid^∧^	12.22	198.06	2.33	1.21	↑	–	0.001	0.460
Glutamic acid^∧^	12.75	246.14	1.41	1.61	↑	↑	0.018	0.001
Ribose^∧^	13.68	73.05	1.66	1.98	↑	↑	0.019	0.002
Palmitic acid	18.71	328.29	1.88	1.32	↑	–	0.001	0.269
Linoleic acid^∧^	21.38	337.27	2.29	1.35	↑	–	0.001	0.395
Oleic acid^∧^	21.48	339.29	2.35	1.34	↑	–	0.002	0.560
Elaidic acid	21.58	339.28	2.16	1.26	↑	–	<0.001	0.444
Stearic acid	21.91	341.30	1.60	1.23	↑	–	0.001	0.210
Unknown-sugar metabolite 1	22.26	217.11	0.52	0.39	↓	↓	0.001	<0.001
Unknown-sugar metabolite 2	23.28	204.11	0.36	0.31	↓	↓	0.003	0.001

*Nine-month old SD rats were sham operated or ovariectomized. At 12 week after ovariectomy, the rats were vehicle treated for 12 weeks. Log_2_ transformation of normalized ion abundance prior to One-way ANOVA with Tukey HSD's post-hoc test*.

∧ *Identified with authentic standards: ↑ upregulated vs. OVX group; ↓ downregulated vs. OVX group; –, no significant change*.

Lipid metabolism disorder frequently accompanies with postmenopausal osteoporosis. As showed in Tables 1, 2, estrogen deficiency resulted in increased body fat deposition and decreased lipid metabolites. UPLC/MS analysis revealed that lysophosphatidylcholine (lysoPC) (20:4), lysoPC (22:6), lysoPC (22:5), lysoPC (22:4) lysoPC (18:0), docosahexaenoic acid (DHA), arachidonic acid, linoleic acid, and oleic acid in OVX group were down-regulated. In addition, the level of steroid metabolites like deoxycortisol were found to be significantly decreased in OVX group owing to the removal of ovaries. Our results clearly indicated that the OVX model was established successfully and provided a base for studying the role of SWR on bone parameters in OVX rats.

**Figure 3:**
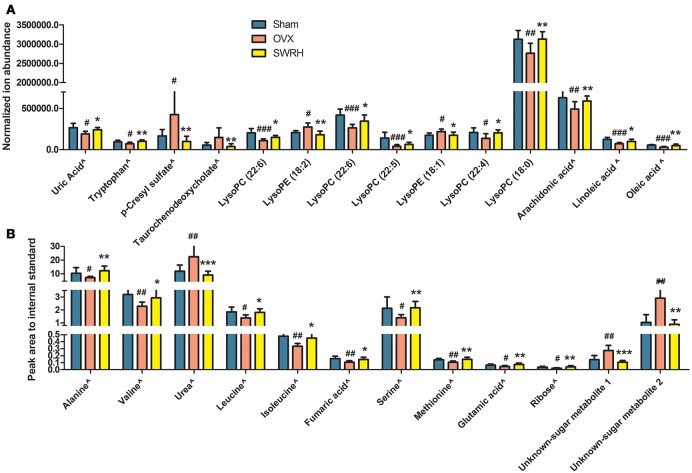
Relative quantities of endogenous metabolites significantly changed after SWRH treatment. Bars represent the mean relative levels and standard deviations. **(A)** UPLC-MS metabolites; **(B)** GC-MS metabolites. ^∧^Identified with authentic standards. ^#^*P* < 0.05, ^*##*^*P* < 0.01, ^*###*^*P* < 0.001 OVX vs. Sham; *P < 0.05, **P < 0.01, ***P < 0.001 SWRH vs. OVX.

Based on the fact that the present animal study clearly demonstrated the protective effects of SWRH on bone properties, multivariate statistical analysis was carried out to characterize the endogenous metabolites that are associated with the efficacy of SWRH treatment. The relative quantities of 26 target metabolites, such as uric acid, tryptophan, *p*-cresyl sulfate, taurochenodeoxycholate, several lysophosphatidylcholines (lysoPCs) and lysophosphatidylethanolamines (lysoPEs), arachidonic acid, linoleic acid, oleic acid, alanine, valine, urea, leucine, isoleucine, fumaric acid, serine, methionine, glutamic acid, ribose, and two unknown sugar derivatives, in LC-MS and GC-MS were significantly altered by SWRH treatment and their levels in serum were restored to Sham level as shown in Figure 3. The lipid profiles from the metabolomics anlyasis showed that the levels of unsaturated fatty acids, lysoPC (22:5), lysoPC (22:6), lysoPC (22:4), lysoPC (18:0), arachidonic acid (C20:4), linoleic acid (C18:2), and oleic acid (C18:1), in OVX rats were restored by treatment with SWRH. Our observation indicated that SWRH treatment might accelerate the metabolism of lipids from saturated fatty acid to unsaturated fatty acid, which might account for part of the anabolic actions of SWRH on bone.

**Figure 4:**
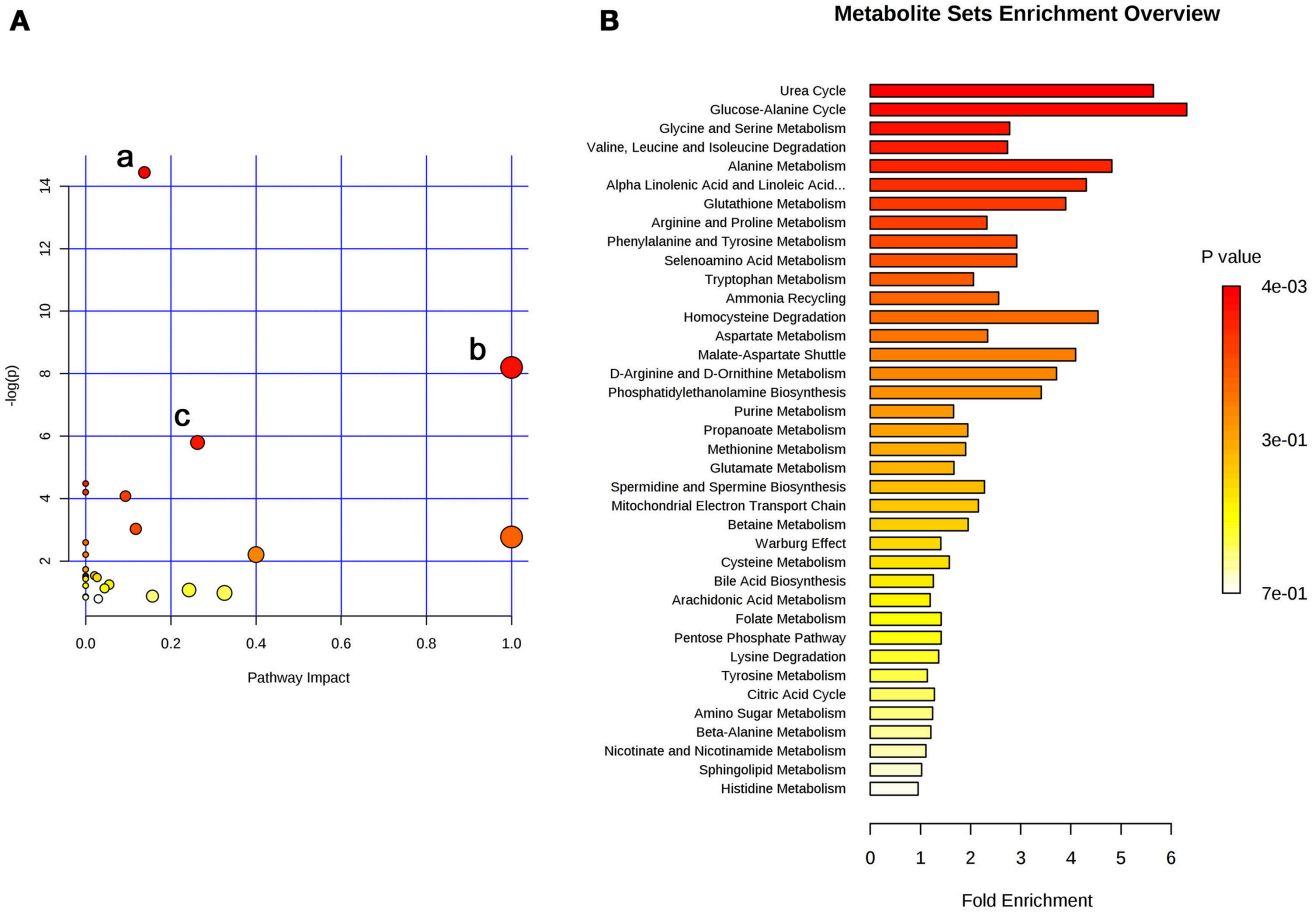
Summary of pathway analysis with Metaboanalyst. **(A)** Pathway impact: (a) aminoacyl-tRNA biosynthesis; (b) valine, leucine, and isoleucine biosynthesis; (c) alanine, aspartate and glutamate metabolism. **(B)** Metabolite sets enrichment overview.

The 26 significant biomarkers were subjected to pathway and enrichment analysis based on Metaboanalyst 4.0 and the associated metabolic pathways were shown in Figure 4. The pathway impact value calculated from pathway topology analysis of >0.1 was filtered out as potential target pathway. Aminoacyl-tRNA biosynthesis (Figure 4Aa), valine, leucine, and isoleucine biosynthesis (Figure 4Ab), and alanine, aspartate and glutamate metabolism (Figure 4Ac) were the most important metabolic pathways affected by treatment with SWRH. The three important metabolic pathways were all related with amino acid metabolism, suggesting that amino acid metabolism might play a major role on the protective effects of SWRH on bone. The metabolite set enrichment analysis in Figure 4B indicated that urea cycle, glucose-alanine cycle, glycine, and serine metabolism, valine, leucine, and isoleucine degradation, and alanine metabolism are the top five metabolite concentrated sets being altered by the treatment with SWRH. All metabolite sets except urea cycle were directly associated with amino acid metabolism, while the urea cycle could convert the toxic ammonia, the product of all amino acid catabolism, to a less toxic urea and ultimately excrete it to urine (Hook et al., 2016). The metabolite set enrichment analysis further confirmed that amino acid metabolism is a key factor involved in the bone protective effects of SWR.

### Validation of significantly restored metabolites by SWR treatment

**Figure 5:**
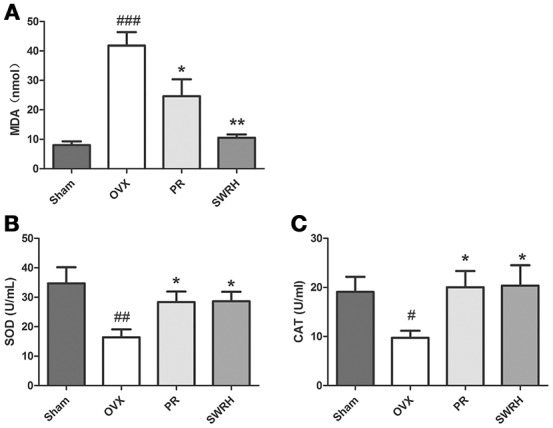
Effects of SWRH on anti-oxidative system in aged Ovariectomized rats. Nine-month old SD rats were subjected to the following treatment for 12 weeks started at 12 week after ovariectomy: Sham, Sham-operated, vehicle-treated; OVX, ovariectomized, vehicle-treated; PR, ovariectomized, Premarin-treated (0.13 mg/kg); SWRH, ovariectomized, high dose SWR-treated (228 mg/kg). **(A)** Malondialdehyde (MDA); **(B)** Superoxide dismutase (SOD); **(C)** Catalase (CAT). Results were expressed as mean ± SEM. ^#^*P* < 0.05, ^*##*^*P* < 0.001, ^*###*^*P* < 0.0001 vs. control; *P < 0.05, **P < 0.01 vs. OVX.

The significantly resorted metabolites, uric acid and *p*-cresyl sulfate by SWRH compared to OVX rats (Figure 3) are related to antioxidative system. In order to confirm if the effects of SWR was associated to antioxidation, the levels of malondialdehyde (MDA) as a wildly used biomarker of oxidative stress, and the antioxidative enzymes of superoxide dismutase (SOD) and catalase (CAT) (Effendy and Shuid, 2014) in serum were measured. Figure 5 shows that OVX dramatically induced MDA by five-fold, while Premarin- and SWRH significantly suppressed the increase of MDA in serum of OVX rats (Figure 5A). The serum levels of SOD (Figure 5B) and CAT (Figure 5C) were significantly decreased in OVX group when compared to Sham group and were significantly increased in OVX rats treated with Premarin and SWRH.

**Figure 6:**
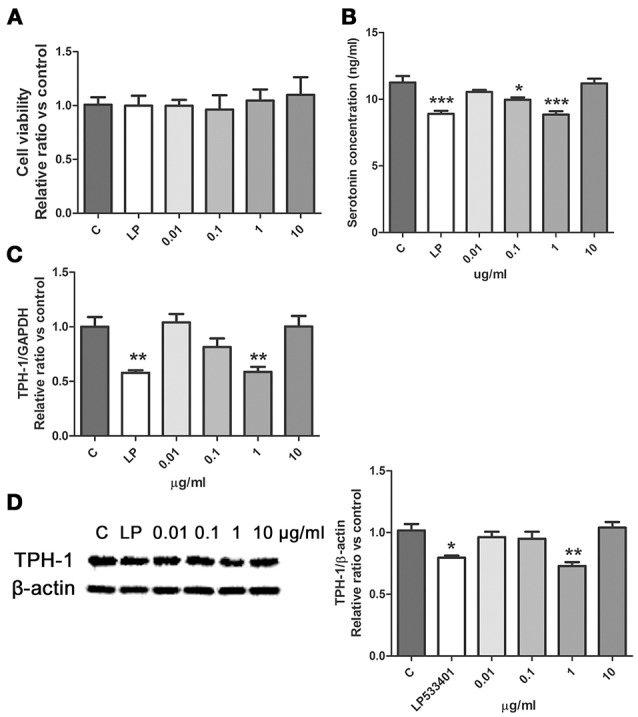
Effects of SWR on serotonin synthesis in RBL-2H3 cells. Cells were treated with control, LP533401, SWR (0.01–100 μg/ml) for 48 h. **(A)** Cell viability; **(B)** Serotonin concentration; **(C)** Relative gene expression of TPH-1 and GAPDH; **(D)** Protein blot and relative intensity of TPH-1 and β-actin. Results were obtained from three independent experiments and expressed as mean ± SEM. *P < 0.05; **P < 0.01; ***P < 0.001 vs. control.

SWR significantly restored the level of tryptophan in serum of OVX rats (Figure 3A), suggesting that tryptophan metabolism might be involved in the effects of SWR on bone. Serotonin is one of the important metabolites of tryptophan and recent study reported that the gut-derived serotonin suppressed bone formation by inhibiting osteoblast proliferation via 5-hydroxytryptamine_1B_ receptor (5-HT_1B_R) (Michalowska et al., 2015). Tryptophan hydroxylase (TPH-1) is the rate-limiting synthetic enzyme of serotonin synthesis located on enterochromaffin cells (Spohn and Mawe, 2017). Thus, in an attempt to explore the potential role of gut-derived serotonin synthesis in mediating the actions of SWR, the effects of SWR on TPH-1 high expressing cells (rat RBL-2H3 cells) were studied. As showed in Figure 6, the inhibitor LP533401 and all concentration of SWR did not affect cell viability, while LP533401 and SWR (at 1 μg/ml) significantly decreased the synthesis of serotonin in RBL-2H3 cells. In addition, SWR at 1 μg/ml significantly decreased TPH-1 mRNA and protein expressions in RBL-2H3 cells (Figures 6C,D). However, the effects of SWR on serotonin synthesis were not in a dose-dependent manner. SWR is a mixture with multi-components and the actions of SWR on inhibiting serotonin synthesis is the combined results of synergistic effects and antagonistic effects of all compounds. Therefore the effects of SWR might be not in dose dependent manner.

## Discussion

The present study demonstrated that the lignan-rich fraction from Sambucus Williamsii Ramulus could alter endogenous metabolic pathways in aged ovariectomized rats and such changes might be associated with its bone protective effects. The novel findings of the present study include: (1) SWR attenuated bone loss, improved bone micro-architecture and increased bone strength in aged rat with established estrogen deficiency conditions for 12 weeks (Table 1); (2) the results of metabolomics revealed that 26 metabolites altered in OVX rats were restored to the Sham level upon treatment with SWRH and these metabolites are associated with the biosynthesis of aminoacyl-tRNA, the biosynthesis of valine, leucine, and isoleucine, and the metabolic pathways for alanine, aspartate and glutamate (Figures 3, 4); (3) amino acid and lipid metabolism appeared to be the major metabolic patterns being affected by SWR and might account for its protective effects on bone (Figures 3, 4); (4) SWR was found to exert anti-oxidative effects *in vivo* and inhibit serotonin synthesis *in vitro* (Figures 5, 6).

**Figure 7:**
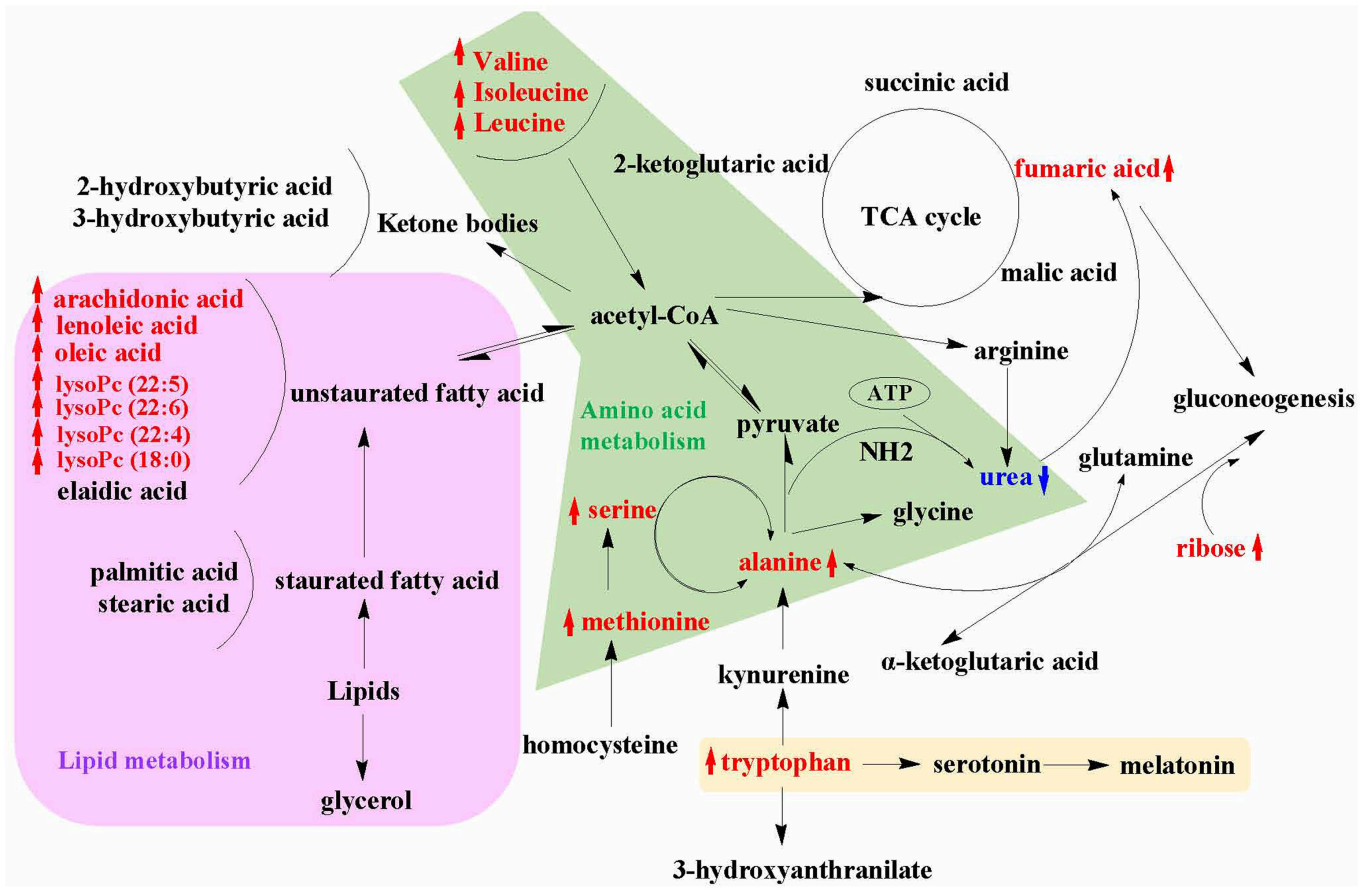
An overview of the metabolic pathways related to SWR treatment. The notations are as follows: red arrow ↑ upregulated vs. OVX group; blue arrow ↓ downregulated vs. OVX group. Purple shade, lipid metabolism; green shade, amino acid metabolism; yellow shade, tryptophan metabolism.

An overview of the metabolic pathways related to SWR treatment were summarized in Figure 7. The same pattern metabolites were accumulated with same color shade. It is obviously showed that lipid and amino acid metabolisms are the two major metabolic pathways that involved in the bone protective effects of SWR. Tryptophan metabolism is also a key metabolic pathway related to the actions of SWR.

It is well-known that lipid metabolism has a close relationship with bone metabolism. The body fat percentage is positively associated with osteoporotic fracture, which leads to the emerging of an effective alternative tool for screening and assessing risk of osteoporosis by estimating body fat percentage from age, height, and weight (Wyshak, 2010). Biochemical analysis showed that higher level of lipids in blood is accompanied with the osteoporotic postmenopausal women (Li et al., 2015), while lipid-lowering drug, such as statin, could increase bone mineral density in patients and in rodents (Parhami et al., 2000). The impairment of lipid on bone is due to the formation of reactive oxygen species (ROS) by saturated fatty acid peroxidation (Cnop et al., 2001). Increased free saturated fatty acid enhance ROS production, and accumulated ROS leads to cells lipotoxicity (Pino and Rodríguez, 2017). On the contrary, unsaturated fatty acids were reported to exert protective effects on bone by inhibiting osteoclast differentiation and osteolytic activity, especially for n-6 and n-3 polyunsaturated fatty acid such as arachidonic acid, linoleic acid, α-linolenic acid, ecicosapentaenoic acid, and decosahexaenoic acid (Pino and Rodríguez, 2017). All these unsaturated fatty acids inhibited RANKL-induced osteoclast differentiation and decreased the expression of osteoclastogenic mRNAs and proteins (Jan et al., 2014; Song et al., 2017). In our results, SWRH treatment restored the level of arachidonic acid (20:4), linoleic acid (18:2), and oleic acid (C18:1) in OVX rats, suggesting that SWRH might exert bone protective effects by inhibiting osteoclastogenesis process. This deduction was consisted with the up-regulated gene expression of OPG/RANKL in RT-PCR assay. Lysophosphatidylcholines (lysoPCs) are the main phospholipid components of low-density lipoprotein. Zhang et al. (2018) reported that several lysoPCs of (18:1), (20:5), (20:0), (18:3), (20:1), (16:1), and (17:0) were down-regulated obviously in OVX rats, which is similar with our results that lysoPC (20:4), lysoPC (22:6), lysoPC (22:5), lysoPC (22:4), and lysoPC (18:0) were reduced in OVX rats (Table 1). Furthermore, treatment of SWRH restored lysoPC (22:5), lysoPC (22:6), lysoPC (22:4), and lysoPC (18:0) to normal level (Figure 3A). These findings indicated that lysoPCs might be a key factor for identifying osteoporosis and elucidating the actions of SWRH on bone. Taken together, we can conclude that SWR might protect bone from estrogen deficiency by inhibiting bone resorption via modulating lipid metabolism.

An increased homocysteine level in plasma due to the lack of estrogen in postmenopausal women was reported to be a risk factor for the development of osteoporosis and bone fracture (Koh et al., 2006). Estrogen influences the metabolic transformation between methionine and homocysteine. Methionine restriction is recently reported to result in low bone mass in mice and the reduced in bone mass might be caused by the increase in collagen degradation during methionine restriction [35]. In our rat model, the level of methionine, and serine, which is the substrate of homocysteine elimination, were down-regulated significantly in OVX group. Our outcomes from metabolomics indicated that the levels of both methionine and serine (metabolites in the methionine cycle) were significantly upregulated in OVX rats in response to treatment with SWRH. These results indicated that SWRH might improve bone mass in OVX rats by restoring the alteration of methionine and serine in homocysteine metabolism induced by estrogen-deficiency. Branched-chain amino acid (BCAA) including valine, leucine and isoleucine, is a class of amino acid having aliphatic side-chains with a branch. Although there are no direct evidences for the effects of BCAA on bone, it could improve the proliferation and survival of hematopoietic stem cells by reforming the entire hematopoietic system of bone marrow (Wilkinson et al., 2018), and it could stimulate protein synthesis in skeletal muscle (Kimball and Jefferson, 2006). As showed in our results, treatment with SWRH restored the amino acid levels of alanine, serine and methionine as well as the BCAA to normal level (Figure 3B). All above results indicated that SWR might exert bone-sparing effects by modulating amino acid metabolism.

Previous study by Maggio et al. (2003). demonstrated that plasma antioxidant levels were markedly reduced in aged osteoporotic women. The level of serum uric acid, an end product of purine metabolism and physiologically works as a predominant antioxidant in the blood circulation, was reported to associate with bone mineral density (Ahn et al., 2013). Our LC-MS metabolomics results showed that serum uric acid was significantly decreased in rats upon ovariectomy, while its level was significantly restored to normal level in OVX rats upon treatment with SWRH. *p*-Cresyl sulfate, a metabolite correlated with the development of bone abnormalities in chronic kidney disease (Tanaka et al., 2013), has been shown to contribute to the increase in intracellular production of reactive oxygen species and induced oxidative stress through the activation of JNK/p38 MAPK phosphorylation in osteoblastic cells. Our study of metabolomics showed that the induction of serum *p*-cresyl sulfate level by OVX were suppressed in OVX rats in response to treatment with SWRH. These results indicated that SWRH plays an important role in suppressing OVX-induced increase in oxidative stress in OVX rats. To verify the findings from our metabolomics, serum levels of oxidative stress biomarkers MDA and antioxidant enzymes including SOD and CAT were examined. Our results clearly indicated that SWRH could reduce the oxidative stress and increase anti-oxidative enzymes in OVX rats and suggest that the bone protective effects of SWR might be mediated through its actions on the anti-oxidative system.

Our LC-MS results in serum showed that the level of tryptophan in OVX rats were significantly downregulated and was restored by treatment with SWRH. Tryptophan, an essential amino acid, is reported to play an important role in bone metabolic diseases (Michalowska et al., 2015). Its level correlated positively with bone mineral density as well as histomorphology of bone formation (Michalowska et al., 2015). Kynurenine is an oxidation product for tryptophan and its level is known to increase with aging and associated with reduced tryptophan level (Braidy et al., 2011). Recent results proved that kynurenine inhibited bone marrow mesenchymal stem cells proliferation, alkaline phosphatase expression and activity and the expression of osteogenic markers like osteocalcin and Runx2 (El Refaey et al., 2015). Thus, it is possible that oxidation of tryptophan was induced during estrogen deficiency, leading to an increase in level of kynurenine and a decrease in tryptophan, thereby inhibiting bone marrow mesenchymal stem cell proliferation and differentiation. In our results, the restored tryptophan level in serum and the up-regulated expressions of osteocalcin and Runx2 upon treatment with SWRH might be due to the inhibiting effects of SWRH on tryptophan oxidation.

Tryptophan is also the precursor for synthesis of serotonin in which the rate-limiting synthetic enzymes of serotonin are tryptophan hydroxylase-1 (TPH-1) and TPH-2 in enterochromaffin cells and neuronal cells, respectively (Spohn and Mawe, 2017). Recent study indicated that serotonin synthesized by brain could promote bone growth by increasing osteoblast proliferation and inhibiting proliferation and differentiation of osteoclasts, while gut-derived serotonin could suppress bone formation by inhibiting osteoblast formation (Lavoie et al., 2017). To determine if SWR could modulate serotonin synthesis, its effects on TPH-1 gene and protein expression in TPH-1 expressing rat RBL-2H3 cells were determined. Our results clearly showed that SWR could suppress the synthesis of serotonin and downregulate TPH-1 mRNA and protein expression in RBL-2H3 cells (Figure 6). Thus, it is possible that SWR might exert bone protective effects by suppressing gut-derived serotonin *in vivo*.

The present metabolomics study provided evidence to support that gut microbiota might participate in the action of SWRH. Specifically, *p*-cresyl sulfate, which is known to be closely associated with gut microflora metabolism, were significantly reduced in OVX rats in response to treatment with SWRH. *p*-Cresol sulfate, the sulfate conjugate of *p*-cresol, is known to be produced by putrefactive bacteria in small intestine from tyrosine through 4-hydroxyphenylacetic acid and has been defined as uremic toxin by the European Uremic Toxin Work Group (Vanholder et al., 2003). Thus, an ability to suppress serum *p*-cresol sulfate in OVX rats suggest that SWRH might be able to alter the activity of microflora in the small intestine. Our study also showed that the level of branch chain amino acids (BCAAs), including valine leucine, isoleucine, were decreased in rats in response to ovariectomy and restored in OVX rats in response to treatment with SWRH (Figure 5). Indeed, BCAAs are known to be derived from gut microbiota transformation and the change in their levels is believed to be a good indicator to reflect the health status of the host and the gut microbial composition (Shoaie et al., 2015). Thus, an increase in serum BCAAs by SWRH in OVX rats further suggest that gut microbial composition might be modified upon long term treatment with SWR extract.

Lignans are the main bioactive chemical class from Sambucus Williamsii Ramulus. By further investigating the change in metabolites composition upon long term treatment, several unique metabolites (m/z: 341.1028, 385.0924, 399.1082, 377.0701, and 473.1452) were found to be only present in the serum from OVX rats treated with SWRH (Figure S1). A preliminary pharmacokinetic study on the lignans was carried out and the in-house results concluded that level of those lignans in serum was too low to be detected in serum obtained from rats treated with high dosage of SWRH. One of the possible explanations for the undetectable level of SWRH phyteochmicals present in rat serum could be attributed to the biotransformation of lignans by gut microbiota before absorption. Further study is certainly necessary to provide a better picture on the role of gut microbiota in improving the bone health after SWRH treatment.

## Conclusion

The present study revealed that lignan-rich fraction from *S. williamsii* significantly altered the levels of metabolites involved in amino acid and lipid metabolism in OVX rats. Some of these metabolites (including methionine, tryptophan, and polyunsaturated fatty acids) are recently known to play a direct or an indirect role in controlling bone mass. In addition, our study provided evidence to support that SWR could modulate anti-oxidative system and serotonin synthesis that might further help to combat against estrogen-deficiency induced oxidative stress and increase in gut-derived serotonin in OVX rats. The change in other metabolites also indicate that SWR could modulate gut microbiota in OVX rats. The current study illustrates a holistic profile of the metabolic changes upon SWR treatment in OVX rats (Figure 7) and provides insight for understanding the unique mechanism of actions of SWR that might be involved in achieving bone protective effects *in vivo*.

## Author contributions

H-HX, DM, X-SY, and M-SW contributed to experimental design. H-HX and XC carried out the animal experiment and all the test *in vivo*. Q-CW generated the cell culture condition and performed all *in vitro* study. T-TS, C-OC, and M-HL contributed to the acquisition and analysis of LC-MS and GC-MS data. DM, X-SY, and M-SW reviewed the manuscript. X-SY and M-SW obtained the funding. H-HX, T-TS, and C-OC wrote the manuscript.

### Conflict of interest statement

The authors declare that the research was conducted in the absence of any commercial or financial relationships that could be construed as a potential conflict of interest.
