# Supplementary material for: A Metabolomics Study on the Bone Protective Effects of a Lignan-Rich Fraction From Sambucus Williamsii Ramulus in Aged Rats
Source: Front Pharmacol. 2018 Aug 21;9:932. doi: 10.3389/fphar.2018.00932 (PMC6110923; doi:10.3389/fphar.2018.00932)

Text S1 UPLC-QTOF/MS and GC-TOF/MS conditions

A 5μl aliquot was injected into a Waters ACQUITY UPLC system. The separation was performed on a Waters ACQUITY UPLC HSS T3 column (2.1 mm × 50 mm, 1.8 μm) with a HSS T3 pre-column (2.1 mm × 5 mm, 1.8 μm, Waters Corporation, Milford, MA, USA). The mobile phase consisted of combinations of A (0.1% formic acid in water, v/v) and B (0.1% formic acid in acetonitrile, v/v) at a flow rate of 0.3 ml/min with elution gradient as follows: 0-1.5 min, 5% B; 2 min, 35% B; 4 min, 50% B; 9 min, 55% B; 12-17 min, 95% B. A 3-min post-run time was set to fully equilibrate the column between injections. Column and sample chamber temperature were set at 40 ^o^C and 5 ^o^C, respectively. Mass spectrometry was performed on a Waters SYNAPT G2 Q-IM-TOF HDMS system (Waters, Milford, USA) operating in an electrospray ion source in negative ionization mode. Nitrogen and argon were used as cone and collision gases. The desolvation gas flow was set to 600 L/h at a desolvation temperature of 400 ^o^C, and the cone gas was set to 40 l/h. The source temperature was set at 120 ^o^C. The capillary voltage in negative ion mode was 2.5 kV. The sampling and extraction cone voltages were 40 V and 4 V, respectively. The scan time was 0.5 s with a 0.024 s interscan delay. Data scan range from m/z 50 to 1000 were recorded in the centroid data format. For accurate mass acquisition, a lock-mass of leucine enkephalin was used and 30 eV trap collision energy, 2.5 kV capillary energy, 40 V cone voltage, monitoring for negative ion mode ([M-H]^−^: m/z 236.1035, 554.2615) to ensure accuracy during the MS analysis. MS/MS analysis was carried out to study the structure of potential biomarkers. In this section, argon was employed as collision gas and the collision energy was set between 5 to 50 eV according to the situation.

For GC-TOF/MS analysis, an Agilent 7890A Gas-Chromatograph (Agilent Technologies Inc., CA, USA) coupled with Waters Micromass GCT Premier Time-of-Flight Spectrometer (Waters, Milford, MA, USA)) equipped with an Ailgent HP-5MS capillary column (30 m × 250 µm i.d, 0.25 µm) was used for the metabolites analysis. 1 µl was injected in the GC-TOF/MS with splitless mode. All injections were carried out using an Agilent 7683 Series autosampler. The column temperature was held at 80 ^o^C for 2 min isothermally and then raised by 10 ^o^C / min to 180 ^o^C, 5 ^o^C / min to 240 ^o^C, finally at a rate of 10 ^o^C / min to 290 ^o^C and held there for 6 min isothermally. The inlet and ion-source temperatures were 270 ^o^C and 230 ^o^C, respectively. Helium was the carrier gas at the constant flow rate of 1 ml/min. The electron impact ionization in positive mode was used and operated at 70 eV. The mass spectrometer was operated under full scan mode in the *m*/*z* range 50-600 with 0.4 s per scan cycle time and 0.38 sec scan duration. Dynamic range enhanced mdoe were selected.

Table S1 Primer used for real-time RT-PCR

| Primer | Accession No. | Sequence (5'-3') | Tm (°C) |
| --- | --- | --- | --- |
| ALP | NM_013059 | F : GCAAGGACATCGCCTATCAG | 53 |
|  |  | R : AGTTCAGTGCGGTTCCAGAC |  |
| OCN | NM_013414 | F: CACAGGGAGGTGTGTGAG | 56 |
|  |  | R: TGTGCCGTCCATACTTTC |  |
| Runx2 | NM_001278484 | F: TAACGGTCTTCACAAATCCTC | 56 |
|  |  | R: GGCGGTCCAGAGAACAAACTA |  |
| OPG | NM_012870 | F : GTTCTTGCACAGCTTCACCA | 55 |
|  |  | R : AAACAGCCCAGTGACCATTC |  |
| RANKL | NM_057149 | F : CATCGGGTTCCCATAAAGTC | 55 |
|  |  | R : CTGAAGCAAATGTTGGCGTA |  |
| GAPDH | NM_017008 | F: AGTCTACTGGCGTCTTCAC | 55 |
|  |  | R: TCATATTTCTCGTGGTTCAC |  |
| TRAP |  | F: ACCGCCTACCTGTGTGGGCA | 64 |
|  |  | R: CCATGAAGTTGCCGGCCCCA |  |
| Ctsk |  | F: CGCCAVGGCAAAGGCAGCTAA | 64 |
|  |  | R: CGGGTCCTACCCGAGCCACT |  |

Runx2, runt-related transcription factor 2; ALP, alkaline phosphatase; OCN, osteocalcin; TRAP, tartrate-resistant acid phosphatase; Ctsk, cathepsin K; OPG, osteoprotegerin; RANKL, receptor activator of nuclear factor κB ligand; GAPDH, glyceraldehydes 3-phosphate dehydrogenase.

Fig. S1 (A) Typical UPLC-QTOF-MS extracted ion chromatograms of metabolites mainly or only detected from (A) SWC-treated group but not in (B) OVX group under negative ESI mode; (C) Heatmap of Metabolites in four groups; (D) mass fragmentation pattern and predicted molecular formulae. Unidentified molecular formulae.
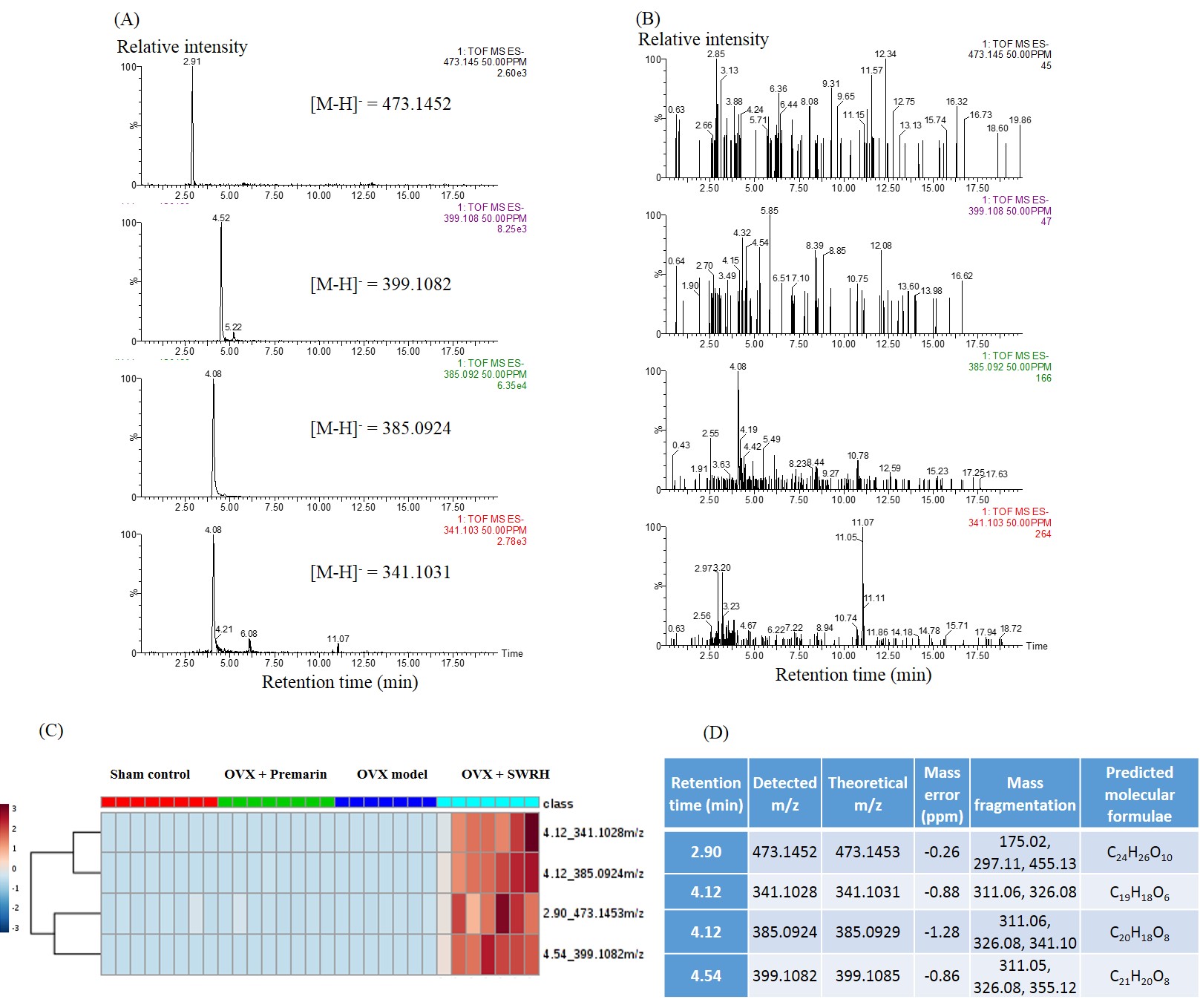

Supplement: Supplementary file 1 [file Data_Sheet_1.docx]
